# Interplay Between SOX9, Wnt/β-Catenin and Androgen Receptor Signaling in Castration-Resistant Prostate Cancer

**DOI:** 10.3390/ijms20092066

**Published:** 2019-04-26

**Authors:** Namrata Khurana, Suresh C. Sikka

**Affiliations:** 1 Department of Internal Medicine-Medical Oncology, Washington University in St. Louis Medical Campus, 660 S Euclid Ave, St. Louis, MO 63110-1010, USA; nkhurana@wustl.edu; 2 Department of Urology, Tulane University School of Medicine,1430 Tulane Avenue, New Orleans, LA 70112, USA

**Keywords:** prostate cancer (PCa), castration-resistant prostate cancer (CRPC), androgen receptor (AR), SOX9, Wnt/β-catenin, sulforaphane (SFN), curcumin (CUR)

## Abstract

Androgen receptor (AR) signaling plays a key role not only in the initiation of prostate cancer (PCa) but also in its transition to aggressive and invasive castration-resistant prostate cancer (CRPC). However, the crosstalk of AR with other signaling pathways contributes significantly to the emergence and growth of CRPC. Wnt/β-catenin signaling facilitates ductal morphogenesis in fetal prostate and its anomalous expression has been linked with PCa. β-catenin has also been reported to form complex with AR and thus augment AR signaling in PCa. The transcription factor SOX9 has been shown to be the driving force of aggressive and invasive PCa cells and regulate AR expression in PCa cells. Furthermore, SOX9 has also been shown to propel PCa by the reactivation of Wnt/β-catenin signaling. In this review, we discuss the critical role of SOX9/AR/Wnt/β-catenin signaling axis in the development and progression of CRPC. The phytochemicals like sulforaphane and curcumin that can concurrently target SOX9, AR and Wnt/β-catenin signaling pathways in PCa may thus be beneficial in the chemoprevention of PCa.

## 1. Introduction

Prostate cancer (PCa) is the second cause of cancer in men and the fifth cause of cancer-related mortality in men worldwide [1]. Prostate-specific antigen (PSA) testing, along with a digital rectal exam (DRE), was approved by the Food and Drug Administration (FDA) for screening asymptomatic men for PCa [2]. PSA is a glycoprotein produced by prostate epithelial cells and has been employed as a primary screening marker for PCa since 1988. PSA levels in the blood are also used for evaluating the effect of cancer treatments and detecting relapse of cancer after initial therapy. Although PSA testing is very sensitive for screening patients with abnormal tumor growth, it is not very specific as PSA levels can even increase with the age and size of the prostate gland and therefore does not necessarily confirm a tumor. Gleason score (GS) is the most extensively used method for grouping tumors [3]. The GS ranges from 2–10; where GS ≤ 6 signifies slow growing and androgen responsive tumors and GS > 7 signifies androgen resistant and invasive metastatic tumors. Tumor-Node-Metastasis (TNM) model is used for tumor staging i.e., extent of disease based on tumor size (T), lymph node invasion (N) and metastasis (M) [4]. The combination of PSA levels, GS and TNM staging is utilized for evaluating disease burden and results of the treatment therapy [5]. Prostatectomy or surgical removal of the prostate is the most extensively used treatment for early stage PCa when the tumor is limited to prostate [6]. Radiotherapy can be either used as an alternative to surgery or along with surgery to treat early stage PCa. Hormone therapy also known as androgen deprivation therapy (ADT) and lastly chemotherapy is given to the patients for whom prostatectomy or radiotherapy fails [7].

## 2. Androgen Receptor Signaling in Prostate Cancer

**Figure 1:**
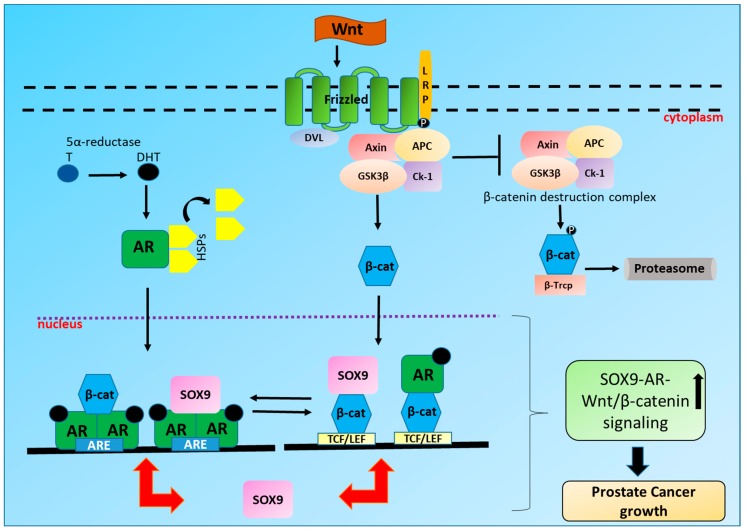
Crosstalk between SOX9, Wnt/β-catenin and Androgen receptor (AR) in Prostate cancer (PCa): On binding with androgen 5α-dihydrotestosterone (DHT); a reduced form of testosterone (T), AR dissociates from heat shock proteins (HSPs), translocates to the nucleus where it dimerizes and binds to the androgen response elements (AREs) of AR dependent genes including SOX9. SOX9 can also in turn bind to AR on AREs leading to the increased transcription and translation of SOX9 and other AR regulated genes like PSA and TMPRSS2. On the other hand, Wnt ligand binds to frizzled (Fz)receptor, inhibiting axin destruction complex leading to the translocation of β-catenin in the nucleus thus resulting in the transcription and translation of T cell factor/lymphoid enhancer factor (TCF/LEF) target genes including SOX9. SOX9 can also bind to β-catenin in the nucleus leading to the increased transcription and translation of TCF/LEF target genes. Additionally, β-catenin and AR signaling can activate each other. Also, β-catenin and AR by acting as a co-activator for each other can modulate both β-catenin and AR signaling in PCa.

Androgen receptor (AR) signaling is crucial not only for the development of prostate gland [8] but also for the initiation, progression and transition of PCa to castration-resistant prostate cancer (CRPC) [9]. AR therefore remains the principal therapeutic target in PCa [10]. AR comprises of three chief functional domains: The N-terminal domain (NTD), DNA binding domain (DBD) and C-terminal ligand binding domain (LBD). NTD incorporates activation function 1 (AF1) which is constitutively active whereas activation function 2 (AF2) in LBD is ligand dependent [11]. AR through DBD, binds to androgen response elements (AREs) in the promoter region of AR regulated genes like PSA [12] and transmembrane serine protease 2 (TMPRSS2) [13]. In the absence of androgens, AR is found primarily in the cytoplasm with its LBD bound to its heat shock proteins (HSPs) i.e., HSP90, HSP70, HSP27 and HSP56 which are responsible for stabilizing its tertiary structure (Figure 1). Testosterone (T) and 5α-dihydrotestosterone (DHT) are the two principal endogenous androgens. Testosterone is metabolized intracellularly by 5α-reductase to a more active reduced form of testosterone i.e., 5α-DHT [8]. DHT has two-fold more predisposition towards AR and decreases the dissociation rate of AR by five-fold compared to testosterone. After DHT binds to LBD, AR dissociates from HSPs thereby exposing nuclear localization signal [14]. AR translocates into the nucleus, dimerizes and binds to AREs leading to the transcription and translation of target genes after recruitment of several coregulatory proteins comprising of coactivators and chromatin remodeling complexes [15].

As AR signaling plays the most critical role in the development and progression of PCa, ADT or androgen ablation therapy (AAT) via luteinizing hormone releasing hormone (LHRH) analogues or AR antagonists like abiraterone acetate, bicalutamide, enzalutamide and flutamide remains the standard of care for PCa patients. Even though nearly all patients respond to ADT in the beginning, PCa patients ultimately become resistant to even high doses of antiandrogens resulting in CRPC [16]. The main factors which are accountable for the growth of CRPC include AR gene amplification, intratumoral/intracrine production of androgens, AR co-activators overexpression, ligand-independent activation of AR by cytokines or kinases [17,18,19] and most importantly the expression of constitutively active AR variants (AR-Vs) lacking LBD, AR-V7 being the major one [20,21]. Targeting AR either directly or by suppressing synthesis of androgen has been shown to considerably increase the survival of metastatic CRPC patients in randomized phase III studies [22]. Enzalutamide [23] and abiraterone acetate [24] have been shown to increase survival in PCa patients. New therapeutic agents which can directly target AR as well as non-coding RNAs or small interfering RNAs (siRNAs) are being developed to suppress the growth of CRPC [25]. AR-Vs are responsible for the progression of CRPC by providing resistance to AR targeting therapies like abiraterone and enzalutamide [26] and also leading to metastasis [27]. AR-V7 has been shown to be an important prognostic biomarker in CRPC [21,28,29].

AR-Vs have been shown to activate AR-full length (FL) in promoting resistance to ADT [20]. The antiandrogen enzalutamide was able to avert the development of CWR22Rv1 (22Rv1) xenograft tumors more efficiently after AR-V7 knockdown underlining the relevance of targeting both AR-FL and AR-V7 for totally abolishing AR signaling. Therefore, therapeutic agents which can target both AR-FL and AR-Vs are presently being investigated and developed for improvising the therapeutic efficacy in CRPC patients [30]. We recently reported that sulforaphane (SFN), an isothiocyanate derived from broccoli can increase the efficacy of antiandrogens in both androgen-dependent as well as androgen-independent cell lines by degrading both AR-FL and AR-V7 [31,32]. 

Interplay between AR and other signaling pathways in PCa modifies the transactivation activity of AR leading to the early emergence of CRPC [33,34]. The synergistic action of the aberrant expression of AR and other signaling pathways responsible for the proliferation and maintenance of PCa cells leads to more aggressive and anomalous expression of the target genes including transcription factors, cell cycle regulators and proteins vital for cell survival, secretion and lipogenesis [19]. Therefore, targeting crosstalk between AR and other critical signaling pathways in PCa can be a key strategy to hamper the progression of PCa and its transition to CRPC. 

## 3. Wnt/β-Catenin Signaling in PCa

Wingless-related integration site (Wnt)/β-catenin signaling is one of the vital mechanisms responsible for cell proliferation, cell polarity, migration and cell fate determination during embryonic development and maintaining tissue homeostasis [35]. Therefore, mutations in this pathway are frequently associated with human birth defects, cancer and other disorders [36,37].When extracellular Wnt signals are not present, cytoplasmic β-catenin gets phosphorylated by glycogen synthase kinase 3 (GSK3) as part of a destruction axin complex which includes casein kinase 1 (CK1), the tumor suppressor adenomatous polyposis coli gene product (APC) and scaffolding axin proteins [38,39] (Figure 1). After sequential phosphorylation by CK1 and GSK3 on the amino terminal region of β-catenin, β-catenin is recognized by an E3 ubiquitin ligase subunit, β-Trcp resulting in its consequent ubiquitination and degradation by the proteasomes. The continuous abolition of β-catenin averts the translocation of β-catenin into the nucleus resulting in the suppression of Wnt target genes by DNA bound T cell factor/lymphoid enhancer factor (TCF/LEF) family of proteins [40]. Wnt ligands bind to the seven-pass transmembrane frizzled receptors (Fz or Fzd) along with cofactor low density lipoprotein receptor-related protein (LRP). Fz signals to scaffolding protein dishevelled (DVL) leading to the phosphorylation of LRP, thereby activating and recruiting axin complex to the receptors. This leads to the suppression of axin facilitated phosphorylation of β-catenin, therefore stabilizing β-catenin and permitting its translocation into the nucleus. β-catenin then binds to TCF/LEF family of transcription factors in the nucleus thereby regulating the expression of target genes such as c-Myc, TCF-1, cyclin D, immunoglobulin transcription factor-2 (ITF-2) and SOX9 [41,42]. An association has been reported between abnormal Wnt/β-catenin signaling and a multitude of human cancers [43,44] including PCa [45,46,47]. Wnt/β-catenin signaling became a center of PCa studies in the late 1990s and early 2000s [48]. Wnt/β-catenin signaling has been shown to affect proliferation of prostate cells, differentiation and epithelial-to-mesenchymal transition (EMT) transition; all of which are regulators of aggressive and invasive behavior of cancer cells [49]. Many secretory Wnt antagonists which are downregulated in cancer can control Wnt/β-catenin signaling. 

Interestingly, a correlation between the aberrant immunoexpression of β-catenin in PCa cells and high probability of death due to the advancement of the tumor has been reported [50]. The study suggested alterations in immunohistochemical (IHC) staining of β-catenin along with high GS in prostate biopsy tissues as a putative prognostic marker for patients with aggressive PCa. Mutations including missense and deletion mutations in β-catenin were found in 5% of PCa metastatic tumor samples [51]. The mutations in β-catenin signaling pathway are thus implicated in the progression of a subset of PCa [52,53]. Wnt/β-catenin signaling was shown to play a critical role in the progression of prostatic intraepithelial neoplasia (PIN) to prostate adenocarcinoma [54] and induce high grade PIN (HGPIN) in a subpopulation of murine prostate luminal epithelial cells [55]. Approximately 24% of metastatic tumors from CRPC patients from several anatomical sites of autopsy samples were reported to be positive for nuclear localization of β-catenin [56]. An abnormal β-catenin expression was found in 23% of radical prostatectomy specimens compared to 38.8% of metastatic CRPC specimens [57]. β-catenin expression was found to be higher in 20 acinar prostatic adenocarcinomas after anti-androgen therapy in prostatectomy specimens compared to the pretreatment biopsies of the same patient group and high grade matched untreated controls [58]. It was reported that 55% of primary prostate and 85% of PCa metastases to lymph nodes and bone specimens showed cytoplasmic and nuclear expression of β-catenin respectively [59]. Besides tissue culture models, β-catenin was also shown to be involved in PCa progression in several genetically engineered mouse models. An overexpression of active β-catenin was found to be associated with high-grade intraepithelial neoplasia and resistance to castration [45]. Wnt signaling has been reported to play a vital role in the tumor microenvironment of prostate as well [60]. The Wnt proteins secreted by the tumor stroma contribute towards resistance to therapy and also help in the expansion or self-renewal of prostate cancer stem cells (progenitor cells). Although Wnt signaling inhibitors are being tested in phase I clinical trials, they have not been tested in PCa patients yet. 

## 4. Interplay between AR and Wnt/β-Catenin Signaling in PCa

β-catenin has been shown to interact directly with AR in yeast and mammalian two-hybrid assays. The interaction sites were shown to be present in the LBD of AR and armadillo repeats in β-catenin [61,62,63]. β-catenin was recognized as an AR interacting protein from a gonadotropin-releasing hormone neuronal cell library using AR deletion construct as a bait [61]. β-catenin was primarily found in the cytoplasm in the absence of androgen and totally co-localized to the nucleus with AR in the presence of DHT. The effect was specific to AR as other liganded receptors like progesterone, glucocorticoid or estrogen alpha could not translocate β-catenin into the nucleus. Also, AR antagonists like bicalutamide and hydroxyflutamide could not translocate β-catenin thus showing that agonist-bound AR was necessary for translocation. The co-translocation took place with similar kinetics as shown by the time course experiments and was independent of GSK3β, p42/44 extracellular signal-regulated kinase (ERK)/mitogen-activated protein kinase (MAPK) and phosphatidylinositol 3-kinase (PI3K) pathways since inhibitors of these pathways showed no effect. β-catenin on binding with LBD, modulated the transcriptional effects of the p160 coactivator transcriptional mediators/intermediary factor 2 (TIF2) and NTD [62]. The binding was independent of and cooperative with NTD and TIF2. The expression of E-cadherin in E-cadherin null PCa cells redistributed β-catenin present in the cytoplasm to the cell membrane leading to the suppression of AR mediated transcription [63]. This finding suggested that lack of E-cadherin can thus augment the cellular levels of β-catenin in PCa cells which directly leads to more malignant and invasive phenotype of the tumor by increasing AR activity during the development and progression of PCa. 

β-catenin is also one of the three co-activators of AR; the other two being ARA70 and ARA55 [64]. Additionally, β-catenin can also act as a coactivator with ARs having mutations (W741C and T877A) in PCa cell lines. W741C mutation was found in CRPC patients treated with bicalutamide whereas T877A mutation was found in CRPC patients with metastatic lesions in the lymph node. 

The analysis of the crystal structure of β-catenin and the nuclear hormone receptor, liver receptor homolog-1 (LRH-1) protein interaction showed that three important β-catenin residues (Y306, K345, and W383) were involved in the interaction [65]. The mutations in these residues were shown to reduce binding of β-catenin to LRH-1 and also AR. 

β-catenin was shown to form complex with AR and increase the transcriptional activity of AR in PCa cells even in the presence of ligands such as androstenedione and 17β-estradiol [66]. Moreover, β-catenin could also abrogate the inhibitory effect of AR antagonist bicalutamide on AR dependent transcription. In the presence of β-catenin, even the weak adrenal androgen androstenedione could activate transcription of AR similar to the strong ligand, DHT. Three active LEF1/TCF binding sites were shown to be present in the promoter region of the *AR* gene and Wnt signaling was able to augment transcription of *AR* [67]. The human *AR* gene was reported to be a target of LEF1/TCF-mediated transcription itself [68].The overexpression of AR increased the transcriptional activities of Wnt/β-catenin signaling in human PCa cell lines when transiently transfected with AR and various components of Wnt signaling pathway [69]. The concurrent overexpression of AR and activation of Wnt signaling stimulated growth and transformation of PCa cells even at castrated levels of androgen. The mutant forms of AR displayed similar or probably decreased capability to promote β-catenin/Wnt1 signaling which explains high frequency of AR wild type overexpression in CRPC specimens. The chromatin immunoprecipitation (ChIP) assays revealed that Wnt3A can cause recruitment of AR to the promoter regions of Myc and cyclin D1, the well-known downstream targets of Wnt signaling pathway. Wnt signaling caused the recruitment of AR and β-catenin to the promoter and enhancer regions of PSA, a well characterized AR target gene. These data suggested that AR promotes Wnt signaling even at the chromatin level and can promote malignancy of prostate cells in a ligand independent manner through this interaction under castrated levels of androgen. The interplay between AR and β-catenin pathways was also detected in a hollow fiber model under castrate and intact conditions [46]. However, this in vivo study depicted interaction and localization of AR and β-catenin only under castration conditions. 

β-catenin nuclear localization was found in 40.7% of CRPC bone metastases [70]. Additionally, 29.6% specimens displayed both β-catenin and AR positive nuclear staining compared to 11.1% of the specimens which displayed positive β-catenin nuclear staining when AR was unnoticeable suggesting that nuclear β-catenin is present more in AR positive nuclei in CRPC tissues. An overexpression of β-catenin protein was detected in 16 CRPC of 29 matched pairs of hormone naïve PCa (HNPC) and CRPC [71]. β-catenin and nuclear AR protein expression was reported to be statistically significant and correlated in CRPC but not in HNPC. The patients treated with ADT who showed short times to progression of PSA were reported to have higher expression of matrix metalloproteinase-7 (MMP-7) which was positively correlated with β-catenin and AR [72].

One of the recent studies showed that prostatic oncogenic transformation majorly occurs in luminal epithelial cells by the aberrant androgen and β-catenin signaling [73]. Most importantly, this study depicted a synergistic effect of AR and β-catenin on the development and progression of PCa identifying a new mechanism for dysregulation of AR and β-catenin expression which is distinct from that of AR or β-catenin alone. The targeted mutational analysis showed that ternary complex of AR, β-catenin and transcriptional intermediary factor-2 (TIF2)/glucocorticoid receptor interacting protein-1 (GRIP1) maintains higher transcription activity than the complex of AR with either β-catenin or TIF2 [74]. Moreover, each coactivator binds the other to the AR leading to augmented transcriptional activity. Also, GRIP1 and β-catenin augmented the activity of both AR and LEF1 in a synergistic manner by getting recruited to AR and LEF1 driven promoters specifically [75]. Thus, the interaction between β-catenin-GRIP1 signified additional possible crosstalk point between AR and β-catenin/Wnt signaling pathways. Inhibitor of β-catenin and T-cell factor (ICAT), a β-catenin interacting protein was shown to suppress Wnt/β-catenin signaling by binding to β-catenin [76]. ICAT was found to express in the human PCa tissues whereas the expression of ICAT was augmented in xenograft tumors in castrated mice. Most importantly, ICAT and AR formed a tertiary complex with β-catenin which stabilized β-catenin-AR complex resulting in elevated AR facilitated transcription and cell growth. The DEAD(Asp-Glu-Ala-Asp) box RNA helicase p68 (Ddx5) has been shown to be a transcriptional co-activator of AR and is overexpressed in PCa tissues compared to benign tissue [77]. Androgens were required for the interaction between Ddx5 and β-catenin in androgen dependent LNCaP as well as LNCaP AI (androgen independent) cells when grown in androgen ablated conditions [78]. Thus, the recruitment of AR and β-catenin to the promoter of androgen dependent genes for AR facilitated transcription was shown to require the function of Ddx5. AR45 (N terminal truncated variant of AR) [79] was reported to interact with AR-FL and suppress AR transcriptional activity and growth of LNCaP cells [80]. Interestingly, AR45 augmented DHT mediated promoter activity of AR when β-catenin was overexpressed. This finding proposed that AR splicing variants may differentially affect PCa cell growth when β-catenin is overexpressed.

β-catenin was shown to coordinate with the loss of phosphatase and tensin homolog (Pten) in promoting invasive carcinoma [81]. Moreover, the efficacy of inhibitors which can either target Wnt receptor complexes at the cell membrane or inhibit the interaction of β-catenin with LEF1 and AR, in averting the progression of PCa has been shown in preclinical studies [60]. In both the normal and neoplastic prostate cells, nuclear β-catenin eventually augments Wnt signaling as well as the activity of AR both in the presence and absence of androgen [48].

AR was shown to induce translocation of β-catenin into the nucleus in AR expressing LNCaP and non-AR expressing PC3 PCa cells [82]. In the presence of exogenous androgen, AR was able to shuttle β-catenin into the nucleus in a time dependent fashion in these cells. However, in the presence of R1881, a synthetic androgen, discrete and punctuated nuclear co-localization of AR and β-catenin was observed. Interestingly, androgen facilitated transport of β-catenin was shown to occur through a unique pathway as AR did not interact with APC or GSK3β. DNA/ligand binding regions of AR and the armadillo repeats of β-catenin were the components required for the nuclear translocation of AR/β-catenin complex and binding to one or more AR promoters leading to significant increase in the AR transcriptional reporter activity as well as nuclear accumulation of β-catenin. DNA binding assay showed that β-catenin can bind to the probasin promoter in an AR dependent manner.

Lee et al. reported that a small molecule inhibitor of β-catenin activity in the nucleus known as C3 can suppress both AR and β-catenin signaling in PCa [83]. Interestingly, C3 suppressed PCa cell growth by disrupting interactions between both β-catenin/AR and β-catenin/T-cell factor suggesting that AR and T-cell factor have overlapping binding sites on β-catenin. Treatment with C3 also reduced β-catenin occupancy on AR promoter and decreased the expression of AR and AR/β-catenin target genes. Moreover, treatment with C3 even suppressed binding of AR to target genes along with reduced recruitment of coactivator-associated arginine methyltransferase-1 (CARM-1), an AR and β-catenin cofactor. The methyltransferase activity of CARM-1 is essential for its synergistic coactivator function with β-catenin for the activation of AR facilitated transcription [84]. Of note, C3 suppressed growth of the tumor in a xenograft model in vivo and obstructed the renewal of bicalutamide resistant sphere forming cells, therefore signifying the therapeutic implications of this approach [83]. 

β-catenin acts as an oncogene by disrupting phosphorylation sites at the N terminus which prevents its recognition by ubiquitin ligases and subsequent degradation [85]. Protease calpain was found to be commonly activated in advanced PCa which resulted in β-catenin cleavage near the N terminus removing the phosphorylation sites and producing a 75 kDa protein [86]. This further validates the concept that β-catenin plays a crucial role in activating AR signaling in advanced and androgen independent PCa. β-catenin when activated during development of prostate resulted in epithelial hyperplasia followed by PIN whereas caused HGPIN and continuous prostatic growth after castration in the adult prostate [45]. AR was initially upregulated with the occurrence of epithelial hyperplasia because of the activation of β-catenin but was downregulated later when HGPIN developed. Interestingly, β-catenin activation caused re-expression of Foxa2 in adult prostate which is generally only expressed during embryonic budding of the prostate. The study further supported the role of constitutive activation of Wnt/β-catenin signaling in the growth of the mouse prostate after castration. 

The cell cycle–related kinase (CCRK), a direct AR transcriptional target gene was shown to play a crucial role in hepatocarcinogenesis by upregulating β-catenin/TCF signaling [87,88]. CCRK connected AR and β-catenin/TCF signaling cascades and was responsible for anomalous activation of β-catenin in human hepatocellular carcinoma (HCC) which induced cell cycle progression and tumor formation in both xenograft and orthotopic models. On the other hand, CCRK knockdown suppressed HCC cell growth which was salvaged by constitutively active β-catenin or TCF. AR, CCRK, and β-catenin were found to concordantly overexpress in the tumor cells in primary human HCC tissue samples. This study suggested targeting of AR-CCRK–β-catenin–positive regulatory circuit as a therapeutic strategy in HCC and other male prevalent cancers. Androgens were shown to activate β-catenin/Wnt signaling in AR positive bladder cancer cells (BCC) [89,90]. In the presence of androgens, AR and β-catenin were shown to co-express in the nuclei of BCC and form a complex with TCF leading to the progression of bladder cancer [90]. This study proposed ADT as a prospective therapeutic approach in the treatment of bladder cancer. Targeting AR-β-catenin signaling axis can thus prove to be beneficial in other male predominant cancers besides PCa.

## 5. SOX9 

SOX9 is a member of the SOX [Sex-Determining Region Y(Sry)-related high-mobility group (HMG) box] family of HMG DNA-binding domain transcription factors [91] which plays a vital role in the embryogenesis including the early development of the prostate gland [92]. It is required in the development of ventral prostate and differentiation of proper anterior prostate [93]. An association was found between elevated levels of SOX9 and the prostate epithelia from the first stages of development of bud from the urogenital sinus. Lack of SOX9 was associated with the reduction in proliferation in the epithelia and loss of expression of structural genes specific to prostate bud development in the ventral prostate. Several studies in PCa cell lines have shown the clinical significance of SOX9 in PCa. In xenograft models, SOX9 was found to augment proliferation and invasion of prostate cell lines [94,95]. High levels of SOX9 were found to be correlated with hormone refractory cancer in human tissue samples [94,96]. Also, dysregulation of SOX7, SOX9 and SOX10 was reported to be linked with aggressiveness of PCa [97]. SOX7 and SOX9 were suggested as prospective prognostic markers of PCa. Most importantly, this study reported downregulation of SOX7 and upregulation of SOX9 as significant mechanisms for the progression of CRPC.

In genetic models of PCa, e.g., transgenic adenocarcinoma of the mouse prostate (TRAMP) and Hi-Myc, deletion of SOX9 prevented the initiation of cancer [98]. The expression profiling of prostate epithelial cells lacking SOX9 showed that SOX9 is able to regulate numerous cytokeratins and cell polarity/adherence due to its role in the initiation of prostate development. One of the recent studies reported SOX9 as the driver of aggressive and invasive PCa [99]. Elevated expression levels of SOX9 were found in the prostate epithelia of genetically modified mice with loss of Pten resulting in metastasis and extremely invasive phenotype. This was further corroborated by in vitro models which showed that SOX9 functions as a fundamental regulator of several processes which cumulatively promote progression of the tumor. SOX9 was shown to promote cell lineage plasticity resulting in cells attaining properties of basal stem cells and also rise in proliferation. Increased expression of SOX9 resulted in the changes in cytoskeleton and adhesion, EMT as well as deposition of extracellular matrix which are traits of highly aggressive and invasive cells. Interestingly, SOX9 mediated invasive phenotype was found to be independent of androgen levels in castrated mice. 

SOX9 was shown in vivo to be highly expressed during fetal prostate development by the expansion of epithelial cells into the mesenchyme, signifying SOX9 can lead to invasive growth in PCa [95]. The overexpression of SOX9 in LNCaP PCa xenografts augmented growth, invasion and angiogenesis. On the other hand, SOX9 inhibition by short hairpin RNA (shRNA) suppressed the growth of 22Rv1 PCa xenografts. This study corroborated the role of SOX9 in the development and maintenance of normal prostate and suggested that this role can add to tumor growth and invasion of PCa. The proliferation as well as the migration abilities of PC-3 cells were considerably suppressed when SOX9 gene was silenced by siRNA in these cells [100]. The expression of the *SOX* gene was found to be higher in PCa tissues than in benign prostatic hyperplasia tissues. Furthermore, a positive correlation was found between SOX9 and GS in PCa patients suggesting the therapeutic and prognostic potential of SOX9 in PCa patients. The epithelial cells at the early stages of prostate epithelia were shown to have an elevated expression of SOX9 which correlated with all the stages of neoplastic progression in Pten and Nkx3.1 mutant mice [101]. The overexpression of SOX9 in prostate epithelia increased cellular proliferation without causing hyperplasia in genetically modified mice. However, overexpression of SOX9 induced early HGPIN in mice heterozygous for the conditional mutant allele of Pten. Conversely, lack of SOX9 in prostate epithelia reduced proliferating cells in normal as well as homozygous Pten mutant mice having prostate neoplasia. Furthermore, SOX9 expression was found to be linked with increasing GS as well as higher Ki67 staining in a cohort of 880 human prostate cancer samples. This study recognized SOX9 as a part of developmental pathway, which gets reactivated in prostate neoplasia regulating proliferation of PCa cells. It also suggested that SOX9 can lead to tumorigenesis in certain genetic contexts. A correlation was found between high expression of SOX9 in residual tumor and early relapse of PCa in a neoadjuvant clinical trial of a combination of androgen deprivation with docetaxel and estramustine [102]. ZBTB7A (Zinc Finger and BR-C, ttk and bab (BTB) Domain Containing 7A) was shown to have a crucial tumor suppressor role in the prostate by physically interacting with SOX9 and functionally antagonizing its transcriptional activity on genes involved in invasion of tumor cells [103].

## 6. SOX9 and AR Signaling

SOX9 was shown to bind specifically to AR DBD glutathione S-transferase fusion proteins and this binding was dependent on a short peptide proximately COOH-terminal to DBD, which is needed for binding between HMG proteins and steroid hormone receptors [94]. Exogenous SOX9 when expressed at high non-physiologic levels inhibited expression and activity of AR whereas led to an increase in protein expression of AR at lower levels. More importantly, SOX9 when downregulated by siRNA in PCa cells suppressed endogenous protein levels of AR without decreasing the message levels showing that regulation of AR expression by SOX9 is through a posttranscriptional mechanism. Moreover, downregulation of SOX9 by siRNA also suppressed proliferation of PCa cells and increased p27 expression; clearly demonstrating the role of SOX9 in supporting PCa cell growth. This study suggested that AR is one of the SOX9 regulated proteins in PCa cells. It also proposed that SOX9 supports the proliferative potential of basal cells and luminal epithelium in the normal prostate whereas in PCa, SOX9 may be crucial to support proliferation and growth of PCa cells independent of basal cells.

The transcription factor ERG (erythroblast transformation-specific (ETS)- related gene) is placed under the control of AR regulated TMPRSS2 promoter in *TMPRSS2: ERG* gene fusions which take place in nearly 50% of PCa. This leads to increased expression level of androgen induced ERG [104]. The overexpression of SOX9 caused neoplasia and promoted invasion of tumor in prostate of murine models in the same way as ERG [105]. The reduction of SOX9 in VCaP cells significantly suppressed invasion and growth in vitro and in vivo, thus showing that SOX9 is a crucial downstream effector of ERG. SOX9 was regulated indirectly by ERG by opening a cryptic AR-regulated enhancer in the *SOX9* gene. Most importantly, this study showed that AR is redirected to genes such as SOX9 which are not generally androgen induced by ERG and thus recognized SOX9 as a crucial downstream effector of ERG in PCa positive for TMPRSS2: ERG fusion. The findings thus established a crucial role for SOX9 in PCa and proposed that the normal function of SOX9 in the development of prostate can be reactivated in PCa for driving invasive growth. The NLR (NACHT and Leucine Rich Repeat domain containing protein) related protein NWD1 (NACHT and WD repeat domain-containing protein 1), acting downstream of SOX9 was shown to modify activity of AR by the stabilization of AR protein levels and stimulating the expression of AR co-activator PDEF (prostate-derived ETS factor) [106].

## 7. SOX9 and Wnt/β-Catenin Signaling

SOX-Wnt interactions have been shown to regulate development and disease in a multitude of situations [107]. SOX factors regulate β-catenin/TCF activity by several mechanisms which involve DNA binding, protein-protein interactions, protein stability as well as recruitment of cofactors. Earlier studies have reported that SOX9 expression can be upregulated downstream of Wnt/ β-catenin signaling. SOX9 is induced in colonic crypts by β-catenin/TCF4 [108]. SOX9 was also shown to be induced by Wnt signaling in PCa cells [94]. SOX9 was shown to positively regulate multiple genes of Wnt pathway including the genes encoding Wnt receptors (Fz and LRP family members) and downstream β-catenin effector TCF4 [109]. The expression levels of SOX9 and Wnt pathway components were found to be associated in PCa xenografts and clinical samples. Also, in SOX9 expressing PCa cells, Wnt synthesis inhibitor (LGK974) could reduce Wnt signaling in vitro and growth of tumor in murine xenograft models. The data thus showed that SOX9 can drive PCa by reactivation of Wnt/β-catenin signaling which facilitates ductal morphogenesis in fetal prostate. The expression of SOX9 was increased after treatment with GSK-3β inhibitor and decreased when β-catenin was downregulated by siRNA, signifying that SOX9 in PCa is regulated by Wnt/β-catenin signaling. This study signified a possible mechanism by which Wnt can be reactivated in PCa. The nuclear form of mesenchymal epithelial transition factor (nMET) was found to considerably increase in human CRPC samples [110]. ADT caused induction of nMET which activated both SOX9 and β-catenin to drive the growth of CRPC. The co-immunoprecipitation studies confirmed that SOX9 and β-catenin form a physical complex [111]. The C-terminal transactivation domain of SOX9 was shown to bind to armadillo repeats overlapping the TCF/LEF-binding site within β-catenin in in vitro binding assays.

SOX9 has been shown to be regulated by β-catenin in squamous cell carcinoma 12 (SCC12) cells [112]. SOX9 was induced when constitutively active form of β-catenin was overexpressed whereas it was downregulated with the knockdown of β-catenin. When β-catenin was knocked down, colony forming ability of SCC cells was markedly reduced. Similarly, reduction of the expression of SOX9 by recombinant adenovirus expressing specific microRNA (miR) significantly decreased the colony forming ability of SCC cells. Interestingly, overexpression of SOX9 in cells with β-catenin knockdown partly restored the colony forming potential of SCC cells. The data suggested that SOX9 is a downstream transcription factor of β-catenin and is positively linked with the development of SCC. The overexpression of SOX9 activated Wnt/β-catenin signaling and SOX9-Wnt/β-catenin axis regulated apoptosis of human lung cancer cells [113]. The crosstalk between SOX9 and β-catenin/Wnt signaling was also reported in the progression of gastric cancer [114] and colorectal cancer [115].

## 8. Conclusions

**Figure 2:**
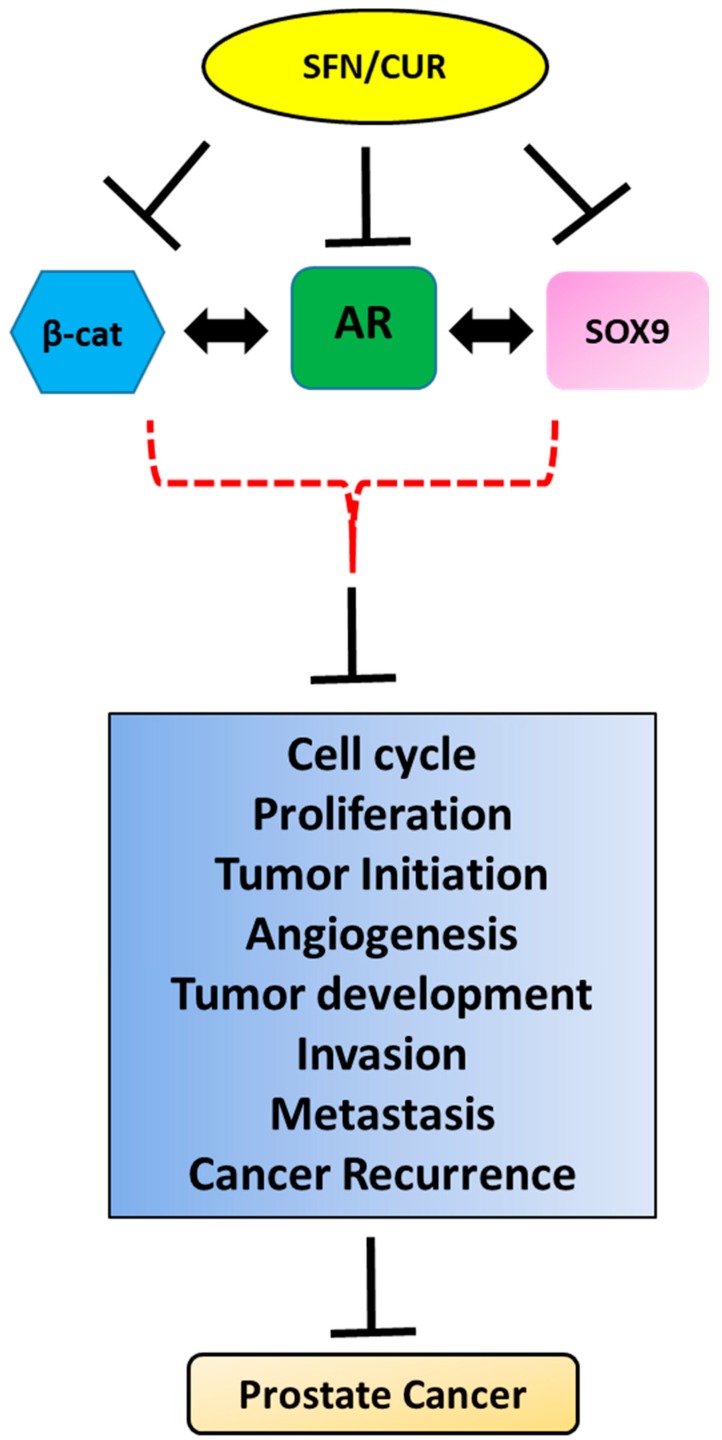
Pleiotropic anti-cancer effects of sulforaphane/curcumin (SFN/CUR): Phytochemicals like SFN/CUR exhibit their anti-cancer effect in PCa by the inhibition of SOX9, Wnt/β-catenin and AR signaling and possibly their crosstalk leading to the overall suppression of tumorigenic parameters of PCa growth.

The crosstalk between SOX9, AR and Wnt/β-catenin signaling in PCa leads to the synergistic aberrant expression of the target genes involved in cell viability, multiplication and differentiation resulting in the early emergence of invasive and aggressive CRPC (Figure 1). Therefore, targeting the SOX9-AR-Wnt/β-catenin signaling axis in PCa, especially CRPC, may be a promising approach for hampering the growth of PCa, both at the initial stage as well as during the later stages of CRPC. Dietary phytochemicals like SFN [116,117] and curcumin (CUR) [118,119] have shown anti-cancer efficacy in a number of PCa studies. SFN has been shown to degrade both AR-FL and AR-V7 in androgen dependent as well as androgen independent PCa cells and potentiate the efficacy of antiandrogens [31,32]. SFN has also been shown to inhibit breast cancer stem cells by phosphorylating β-catenin resulting in its degradation [120]. Furthermore, SFN downregulated SOX9 in ductal carcinoma in situ (DCIS) leading to reduced stem-like cell frequency in vitro and tumor growth in vivo [121]. Similar to SFN, CUR has also been shown to degrade AR [122], downregulate Wnt/β-catenin signaling [123] and SOX9 expression [124]. These findings corroborate the importance of targeting the crucial crosstalk between SOX9, Wnt/β-catenin and AR as a chemo preventive approach in the treatment of PCa (Figure 2). However, dietary phytochemicals like SFN and CUR need to be further characterized for their chemo preventive properties and selective toxicity to cancer cells before their clinical usage as anti-cancer agent alone or in conjunction with conventional treatment regimen.

## Abbreviations

AR	Androgen receptor
PCa	Prostate cancer
CRPC	Castration-resistant prostate cancer
LBD	Ligand binding domain
